# Horticultural Activity Type, Psychological Well-Being, and Fruit and Vegetable Intake

**DOI:** 10.3390/nu12113296

**Published:** 2020-10-28

**Authors:** Yu-Qiao Zhong, Hung-Ming Tu

**Affiliations:** Department of Horticulture, National Chung Hsing University, Taichung 40227, Taiwan; jj5jm3@gmail.com

**Keywords:** indoor plant activities, outdoor plant activities, arts/crafts activities, excursions, Chinese Happiness Inventory, Brief Symptom Rating Scale, horticultural therapy, PLS-SEM

## Abstract

The purpose of this study was to explore the effect of the frequency of participation in horticultural activity types on psychological well-being and fruit and vegetable intake. The study sought to understand the mediating effect of psychological well-being between the frequency of types of horticultural activities and the frequency of fruit and vegetable intake. Convenience sampling was used to collect 400 valid data through a self-administered questionnaire that inquired about the frequency of four horticultural activity types (indoor plant activities, outdoor plant activities, arts/crafts activities, and excursions), the measure of psychological well-being, and the frequency of fruit and vegetable intake. The results showed that a higher frequency of indoor and outdoor plant activity positively affected psychological well-being. Psychological well-being played a partial mediation role between indoor plant activity and vegetable and fruit intake and a full mediation role between outdoor plant activity and vegetable and fruit intake. The plant-related arts/crafts activities and excursions were not associated with psychological well-being or vegetable and fruit intake.

## 1. Introduction

Participating in horticultural activities produces multiple benefits, including improvements in psychological well-being [1,2]. Zhu et al. designed a randomized controlled trial to demonstrate that a horticultural therapy program improved positive and negative emotions in patients with schizophrenia [1]. Ng et al. showed that a horticultural therapy program promoted psychological well-being in older adults [2]. Several studies have pointed out that participating in horticultural activity increases fruit and vegetable intake [3]. For instance, Alaimo et al. demonstrated that participating in a community garden increased the vegetable and fruit intake of urban adults [3]. Heim et al. showed that garden-based experiential learning activities increased fruit and vegetable intake in children [4]. This study focused on the mental health, psychological well-being, and the fruit and vegetable intake of individuals participating in horticultural activities.

Horticultural activities encompass multiple activity types. According to Relf [5], therapeutic tools in horticulture include arts/craft, group activities, excursions, indoor plant activities, outdoor plant activities, and related fields of study. For example, Masuya et al. [6] and Kenmochi et al. [7] mainly planned indoor plant activities in a horticultural activities program, whereas Park et al. [8] mainly planned outdoor plant activities in a horticultural activities program. The program activities can be inducted into four major types from past studies: indoor plant activities, outdoor plant activities, arts/crafts activities, and excursions [9]. Most horticultural activity programs consist of more than one type of activity based on the purpose of the program and the subject’s characteristics [9]. The four main types of activities mentioned above are also common leisure horticultural activities [10,11,12]. However, few studies have explored the benefits of different types of horticultural activities.

Different types of horticultural activities may produce different and similar benefits. For example, Tu et al. [13] showed that four types of horticultural activities (grass doll, kokedama, rocky leaf prints, and herb tasting and smelling) produced different physiological and mental states. Gigliotti et al. [14] showed that the three types of horticultural activities (cooking, crafts, and planting) for persons with dementia produced similar levels of positive affect and engagement. Because the activity programmers need to plan different horticultural activities for varied people and groups, the activity modifications for each horticultural program is critical from the evidence-based research [14]. Understanding the benefits of different types of horticultural activities can help activity programmers plans specific activities that match the participants’ needs. Which types of horticultural activities should be considered to meet the participants’ needs when activity programmers decide to promote mental health, well-being, and fruit and vegetable intake? For example, the frequency of participation in indoor and outdoor plant activities may have benefits for increasing the frequency of fruit and vegetable intake because past studies have indicated that fruit and vegetable intake is associated with the garden-based activities [3,4]. On the other hand, arts/crafts activities and excursions may not be associated with fruit and vegetable intake because most garden-based activities used gardening with nutritional education, cooking, and harvests to improve vegetable intake [15]. Which types of horticultural activities have benefits for mental health and psychological well-being is also an important issue that requires understanding. The first purpose of this study was to explore the effect of the frequency of participation in horticultural activity types on psychological well-being and on the frequency of fruit and vegetable intake.

**Figure 1:**
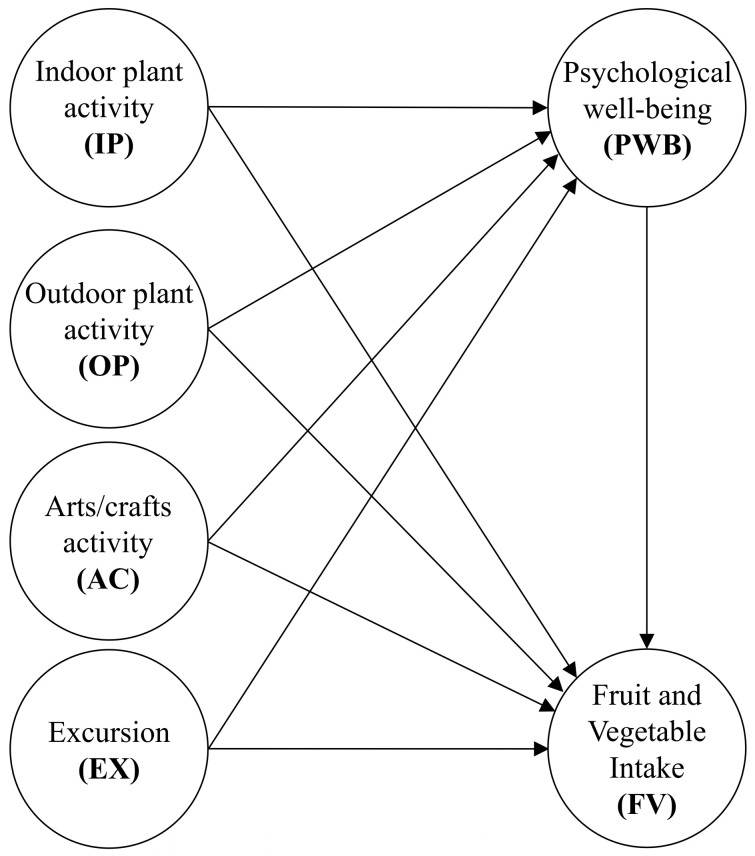
Study framework.

Some studies have indicated that gardening does not affect fruit and vegetable intake [16,17]. Psychological well-being may be a potential mediating variable because positive psychological benefits affect health and nutrition behaviors, so well-being may predict health behaviors regarding fruit and vegetable intake [18]. Few studies have explored the potential mediation variables between horticultural activities and fruit and vegetable intake. Therefore, the second purpose of this study was to explore the mediation effect of psychological well-being between the frequency of four types of horticultural activities and the frequency of fruit and vegetable intake. According to the two purposes of this study, the study framework was determined and is shown in Figure 1.

## 2. Materials and Methods

### 2.1. Independent Variable

The independent variable was the frequency of participation in the types of horticultural activities. The horticultural activities can be generalized into four types based on previous studies [5,10,11,12] and horticultural therapy [1,2,6,7,8,9,19,20,21,22,23,24,25,26,27,28]. These include indoor plant activities, outdoor plant activities, arts/crafts activities, and excursions. Indoor plant activities included planting living plant in pots and using hydroponic cultivation in indoor houses. Outdoor plant activities included planting flowers, vegetables, fruits, and other plants in an outdoor environment. The arts/crafts activities included projects on living and lifeless plants, such as flower design; pressed, dried, and handmade flowers; plant ornaments; and other related activities. Excursions included travel to related horticultural spaces, such as farms, gardens, parks, exhibitions, mountains, and other natural spaces. This study used a 7-point scale to assess the frequency of participation during the last year in the four types of horticultural activities from 1 = absolute infrequency, 4 = regular frequency participation, and 7 = absolute frequency.

### 2.2. Mediating Variable

The mediating variable was psychological well-being. The Chinese Happiness Inventory (CHI) [29] was used to measure subjective well-being. The CHI combines the Oxford Happiness Inventory and the Chinese culture and has demonstrated good reliability and validity to measure Chinese subjective well-being [29]. The original inventory has 48 items. Considering the respondents’ patience, this study used a brief 10-item version of the CHI, which has demonstrated good reliability, to measure overall well-being [30,31,32,33]. The 10-item version consists of six dimensions, including optimism, positive affect, satisfaction with self, physical fitness, achievement at work, and peace of mind. Each item was assessed by the specific four statements. For example, one item of optimism dimension assessed four statements “I’m just messing around” (0 scores), “I like my life” (1 score), “I like my life very much” (2 scores), and “I love my life” (3 scores). The four statements were coded scores as 0 (lowest level of well-being) to 3 (highest level of well-being) for each item [30,31,32,33] to assess the levels of well-being over the last year. Higher scores presented higher subjective well-being. The overall Cronbach’s alpha was 0.93 in this study.

### 2.3. Dependent Variable

The dependent variable was the frequency of fruit and vegetable intake. Food frequency [34] was selected to assess the frequency of fruit and vegetable intake. This study referred to previous research [34,35] and used one item to assess the frequency of vegetable intake and one item to assess the frequency of fruit intake over the last one year on a 7-point scale ranging from 1 (absolute infrequency) to 7 (absolute frequency).

### 2.4. Data Collection

The flower market was a suitable site for collecting participants who take part in various types of gardening activities [10,11,12]. We determined that the largest flower market in Taipei City in Taiwan (the Jianguo Holiday Flower Market) was appropriate for collecting data from 5 January 2020 to 26 February 2020. This study used convenience sampling to capture individuals over 20 years old who participate in horticultural activities and the data were collected through a self-administered questionnaire, including the aforementioned independent variables, dependent variables, and control variables. Finally, 400 valid questionnaires were obtained. Female respondents (53.7%) responded more than male respondents (46.3%) (Table 1). More respondents were 50 to 59 years old (32.5%) or 40 to 49 years old (26.3%). Two-fifths of respondents were 20 to 29 years old (15.0%), 30 to 39 year old (13.3%), or older than 60 (13.0%). At the educational level, four-fifths of respondents had higher education, including university (44.3%), postgraduate (21.5%), and college (18.5%) education. In terms of monthly income, three-fifths of the respondents had less than USD 1666 (TWD 50,000), including less than USD 833 (TWD 25,000) (22.0%) and USD 834–1666 (TWD 25,001 to 50,000) (36.3%).

### 2.5. Data Analysis

The variables’ values of skewness and kurtosis were higher than 1.00 or lower than −1.00 (Table 2), which indicated non-normal data. The Partial Least Squares Structural Equation Modeling (PLS-SEM) method, which can analyze mediation analysis with multiple single-item independent variables in non-normal data [36,37,38] was used. This study used PLS-SEM to analyze the mediation relationship among the frequency participation in types of horticultural activities, psychological well-being, and the frequency of fruit and vegetable intake. Past references have suggested that the PLS-SEM measurement model should match the criteria below to reach internal consistency, convergent validity, and discriminant validity: Cronbach’s alpha > 0.7; composite reliability (CR) > 0.70; average variance extracted (AVE) > 0.50; square root of AVE’s > variables’ correlations; outer loadings > 0.70 and cross-loadings [37,39]. The PLS-SEM structural model should implement the steps below and consider the following criteria: testing collinearity through variance inflation faction (VIF) < 5.0; assessing in-sample predictive power through small (0.25) to large (0.75) *R*^2^ value and out-of-sample predictive power through Q^2^ values > 0.00; estimating effect size through small (0.02) to large (0.35) *f*^2^ and *q*^2^ value; and evaluating statistical significance through 5000 bootstrapped samples [37,39]. The PLS-SEM analyses were performed using SmartPLS 3 software [40]. Determination of the mediation effect was based on the guideline of Zhao, Lynch, and Chen [41] and Hair et al. [37], which explains that the significant indirect and direct effect was partial mediation and only a significant indirect effect plus no direct effect was full mediation in the PLS-SEM.

## 3. Results

The mean of the frequency of participation in indoor plant activities and excursions was 3.89 and 4.22, respectively, indicating regular frequency participation (Table 2). The mean of the frequency of participation in outdoor plant activities and arts/crafts activities was 2.24 and 2.08, respectively, indicating very infrequent participation. The mean of the dimensions of psychological well-being was 1.31 to 1.81, meaning that the respondents had moderate well-being. The mean of the intake frequency of vegetables and fruits was 5.60 and 5.25, respectively, indicating some frequency to high frequency.

For psychological well-being and vegetable and fruit intake, each Cronbach’s alpha, CR value, and AVE value was greater than 0.70, 0.70, and 0.05, respectively (Table 2). Each outer loading was also greater than 0.70 and the cross-loadings. The AVE’s square root was greater than the variables’ correlations (Table 3). The measurement model had sufficient internal consistency, convergent validity, and discriminant validity. In the structural model, each variable’s VIF was less than 5.0, indicating non-problematic levels of collinearity. The in-sample’s predictive power was small for psychological well-being (*R*^2^ = 0.09; adjusted *R*^2^ = 0.08) and the frequency of fruit and vegetable intake (*R*^2^ = 0.11 adjusted *R*^2^ = 0.10). The Q^2^ values were larger than zero, indicating that the out-of-sample predictive power (Q^2^ was 0.05 and 0.07, respectively, for psychological well-being and the frequency of fruit and vegetable intake). The range of *q*^2^ and *f*^2^ effect sizes were 0.00 to 0.04, indicating small effect sizes.

**Figure 2:**
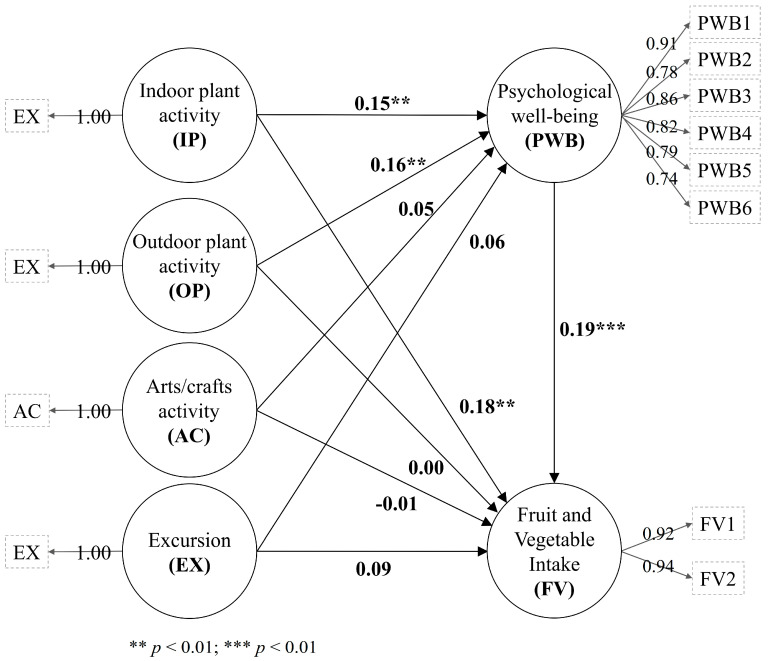
The analysis of PLS-SEM.

The frequency of participation in indoor plant activities had a positive and small direct effect on psychological well-being (*β* = 0.15, BCa 95% CI of 0.03 to 0.25, *f*^2^ = 0.02) (Table 4; Figure 2). Psychological well-being had a positive and small direct effect on the frequency of fruit and vegetable intake (*β* = 0.19, BCa 95% CI of 0.08 to 0.28, *f*^2^ = 0.04). The frequency of participation in indoor plant activities had a positive and small indirect (*β* = 0.03, BCa 95% CI of 0.01 to 0.06) and direct (*β* = 0.18, BCa 95% CI of 0.08 to 0.28) effect on the frequency of fruit and vegetable intake (f2 = 0.03), indicating that psychological well-being had partial mediation.

The frequency of participation in outdoor plant activities had a positive and small direct effect on psychological well-being (*β* = 0.16, BCa 95% CI of 0.06 to 0.25, *f*^2^ = 0.02) and presented a positive and small indirect effect on the frequency of fruit and vegetable intake (*β* = 0.03, BCa 95% CI of 0.01 to 0.06, *f*^2^ = 0.00). The frequency of the participation in outdoor plant activities had no direct effect on the frequency of fruit and vegetable intake (*β* = 0.00, BCa 95% CI of −0.10 to 0.10), indicating that psychological well-being had full mediation. The frequency of the participation in arts/crafts activities and excursions had no direct or indirect effect on psychological well-being or the frequency of fruit and vegetable intake.

## 4. Discussion

The result of this study showed that a higher frequency of indoor plant activities positively affected psychological well-being and the frequency of vegetable and fruit intake. Psychological well-being played a role of partial mediation between indoor plant activities and vegetable and fruit intake. Several studies have indicated that physical activities improve psychological well-being [42]. Outdoor plant activities produce low to moderate intensity physical activities during the process of preparation, planting, and maintenance [43]. The results of this study showed that a higher frequency of outdoor plant activities positively affected psychological well-being. The recent study of He et al. indicated that outdoor plant activities related to do edible vegetations reduced clinical symptoms of schizophrenia in female patients [44]. Interesting, the frequency of outdoor plant activities only had an indirect effect on the frequency of vegetable and fruit intake. The frequency of outdoor plant activities had no direct effect on the frequency of vegetable intake, showing full mediation. Outdoor plant activities completely affected the frequency of vegetable and fruit intake by promoting psychological well-being. Promoting regular public indoor and outdoor plant activities should be considered a useful method for improving psychological well-being and vegetables and fruit intake. The promotion of psychological well-being is an important process in increasing the frequency of vegetable and fruit intake.

Some cross-sectional studies have found that outdoor plant activities are associated with vegetable and fruit intake [3,4]. Some control trials, however, did not find evidence of this relationship [16,17]. Future studies should implement control trials and meta-analysis control trials to explore the relationship between plants activities and vegetable and fruit intake. The lower frequency of participation in outdoor plant activities was a possible factor. Future studies should cover higher frequency participation in outdoor plant activities to explore the effect on the frequency of vegetable and fruit intake. The outdoor plant activities factor was also potentially important because the experience of planting flowers may induce vegetable and fruit intake. Future studies should survey detailed experiences of planting vegetation, fruits, and flowers.

This study indicated that plant-related arts/crafts activities and excursions did not present benefits of psychological well-being. The study period may be a possible factor because this study assessed variables over one year that evaluated long-term horticultural behaviors and benefits. Plant-related arts/crafts activities and excursions may induce short-term benefits but did not affect long-term psychological well-being. The lower frequency of participation in arts/crafts activities was also a possible factor. In addition, plant-related arts/crafts activities and excursions were also not associated with the frequency of vegetable and fruit intake. The reason may be that the arts/crafts activities and excursions do not bring participants directly into contact with the vegetables and fruits but rather allow them to obtain related knowledge.

Some limitations to this study should be understood. First, this study was cross-sectional and cannot determine causality between the variables, although the variables were differentiated into independent and dependent variables. Second, this study did not compare the benefits of different types of horticultural activities because each respondent had different frequencies for the different types of horticultural activities. Third, this study did not add control variables because some studies did not consider the control variables to explore horticultural activities’ benefits on vegetable and fruit intake [3,4]. However, other variables, such as dietary factors, may affect long-term vegetable and fruit intake. Future studies should design control groups and experimental groups with single horticultural activities, using randomized controlled trials. They should consider related control variables to demonstrate causality and compare the benefits of different types of horticultural activities. The method included some strengths in this study. First, using cross-sectional study reduces the investigation time and cost to prove the mediation of psychological well-being. Although, the longitudinal mediation can establish causality. Second, using a cross-sectional study reduces the complexity when dealing with events at different periods in time.

## 5. Conclusions

A higher frequency of indoor and outdoor plant activities positively affected psychological well-being. Psychological well-being played a role of partial mediation between indoor plant activities and vegetable and fruit intake and a role of full mediation between outdoor plant activities and vegetable and fruit intake. Plant-related arts/crafts activities and excursions were not associated with psychological well-being or vegetable and fruit intake. Promoting regular public indoor and outdoor plant activities was a useful method to increase the public’s intake of vegetables and fruit, promoting psychological well-being.

## Figures and Tables

**Table 1:** Descriptive statistics of respondents.

Variables	N	(%)
Sex		
Male	185	46.3
Female	215	53.7
Age (years)		
20–29	60	15.0
30–39	53	13.3
40–49	105	26.3
50–59	130	32.5
60 or older	52	13.0
Education level		
Primary	7	1.8
High school	56	14.0
College	74	18.5
University	177	44.3
Postgraduate	86	21.5
Monthly income		
Less than USD 833 (TWD 25,000)	88	22.0
USD 834–1666 (TWD 25,001–50,000)	145	36.3
USD 1667–2500 (TWD 50,001–75,000)	89	22.3
USD 2501–3333 (TWD 75,001–100,000)	47	11.8
More than USD 3334 (TWD 100,001)	31	7.8

**Table 2:** Internal consistency, convergent validity, convergent validity, and discriminant validity.

Variables	M (SD)		Skewness	Kurtosis	Factor Loading and Cross Loading						Cronbach’s α	Composite Reliability (CR)	Average Variance Extracted (AVE)
					IP	OP	AC	EX	PWB	FV			
IP: indoor plant activity frequency	3.89	(2.17)	0.09	−1.34	1.00	0.24	0.28	0.33	0.22	0.25	1.00	1.00	1.00
OP: outdoor plant activity frequency	2.24	(2.00)	1.40	0.49	0.24	1.00	0.23	0.29	0.22	0.11	1.00	1.00	1.00
AC: arts/crafts activity frequency	2.08	(1.61)	1.49	1.33	0.28	0.23	1.00	0.23	0.15	0.09	1.00	1.00	1.00
EX: excursion frequency	4.22	(1.62)	−0.05	−0.66	0.33	0.29	0.23	1.00	0.17	0.18	1.00	1.00	1.00
PWB: psychological well-being											0.84	0.93	0.86
PWB1: optimism	1.54	(0.74)	0.20	−0.44	0.22	0.19	0.11	0.14	0.91	0.23			
PWB2: achievement at work	1.58	(0.84)	0.03	−0.61	0.17	0.20	0.11	0.15	0.78	0.16			
PWB3: positive affect	1.52	(0.59)	0.47	−0.25	0.19	0.17	0.12	0.12	0.86	0.18			
PWB 4: physical fitness	1.31	(0.76)	0.30	−0.16	0.19	0.21	0.15	0.16	0.82	0.18			
PWB 5: satisfaction with self	1.69	(0.59)	−0.31	0.04	0.13	0.16	0.14	0.09	0.79	0.18			
PWB 6: peace of mind	1.81	(0.77)	−0.12	−0.51	0.18	0.17	0.10	0.17	0.74	0.25			
FV: fruit and vegetable Intake											0.90	0.92	0.67
FV1: vegetable intake frequency	5.60	(1.44)	−0.73	−0.31	0.20	0.10	0.05	0.15	0.21	0.92			
FV2: fruit intake frequency	5.25	(1.55)	−0.50	−0.75	0.26	0.10	0.10	0.18	0.24	0.94			

**Table 3:** Fornell–Larker criterion.

	IP	OP	AC	EX	PWB	FV
IP: indoor plant activity frequency	1.00					
OP: outdoor plant activity frequency	0.24	1.00				
AC: arts/crafts activity frequency	0.28	0.23	1.00			
EX: excursion frequency	0.33	0.29	0.23	1.00		
PWB: psychological well-being	0.22	0.22	0.15	0.17	0.82	
FV: fruit and Vegetable Intake	0.25	0.11	0.09	0.18	0.24	0.93

**Table 4:** Path coefficients of direct effects and indirect effects.

Parameter	Direct Effect			Indirect Effect		
	Coefficient	t Value	BCa 95% CI	Coefficient	t Value	BCa 95% CI
IP -> PWB	0.15 **	2.63	0.03; 0.25			
OP -> PWB	0.16 **	3.08	0.06; 0.25			
AC -> PWB	0.05	1.06	−0.05; 0.15			
EX -> PWB	0.06	1.14	−0.04; 0.18			
IP -> FV	0.18 ***	3.48	0.08; 0.28	0.03 *	2.08	0.01; 0.06
OP -> FV	0.00	0.03	−0.10; 0.10	0.03 *	2.16	0.01; 0.06
AC -> FV	−0.01	0.29	−0.12; 0.08	0.01	0.98	−0.01; 0.03
EX -> FV	0.09	1.71	−0.02; 0.19	0.01	1.08	−0.01; 0.04
PWB -> FV	0.19 ***	3.70	0.08; 0.28			

* *p* < 0.05; ** *p* < 0.01; *** *p* < 0.01; AC: arts/crafts activity frequency; BCa 95% CI: bias-corrected and accelerated 95% confidence interval based on 5000 bootstrapped samples; EX: excursion frequency; FV: fruit and vegetable intake; IP: indoor plant activity frequency; OP: outdoor plant activity frequency; PWB: psychological well-being.
